# “As long as you are married you cannot protect yourself against syphilis”: qualitative exploration of syphilis risk perception and antenatal care seeking among pregnant women in Uganda

**DOI:** 10.1186/s12889-026-26263-1

**Published:** 2026-01-27

**Authors:** Amanda P. Miller, Stephen Mugamba, Bashir Magada, Adriane Wynn, Natalie Saham, William Ddaaki, Emmanuel Kyasanku, Robert Bulamba, Vitalis O. Olwa, James Nkale, Godfrey Kigozi, Fred Nalugoda, Grace N. Kigozi, Alex Daama, Gertrude Nakigozi, Jennifer A. Wagman

**Affiliations:** 1 Division of Epidemiology and Biostatistics, School of Public Heath, San Diego State University, San Diego, CA USA; 2 Africa Medical and Behavioral Sciences Organization, Kampala, Uganda; 3 Division of Infectious Diseases and Global Public Health, School of Medicine, University of California, La Jolla, San Diego, CA USA; 4 Department of Community Health Sciences, Fielding School of Public Health, University of California, Los Angeles, CA USA

**Keywords:** Syphilis testing, Syphilis treatment, Congenital syphilis, HIV, Uganda

## Abstract

**Background:**

Africa accounts for 56% of maternal syphilis and 62% of congenital syphilis cases globally. The high prevalence of syphilis in this region is particularly concerning in the context of a generalized HIV epidemic, as syphilis infection increases potential for sexual transmission of HIV. The present study qualitatively explores perceptions of syphilis risk, transmission, testing and treatment, and experiences accessing antenatal care from the perspectives of currently pregnant people residing in communities across two Ugandan districts: Wakiso and Hoima.

**Methods:**

We conducted focus group discussions (k = 10) with pregnant women (*n* = 82) across six communities. FGDs were audio recorded. Data analysis involved transcription and translation, coding (by two qualitative researchers), and thematic analysis. We promoted trustworthiness through various methodological strategies (e.g. purposive sampling, reflexivity).

**Results:**

Many women described barriers to care seeking at government ANC clinics that centered around poor treatment by providers, long wait times for appointments, high out of pocket costs (due to travel and fees) and the expectation that male partners should accompany them to appointments. This led many to seek their antenatal care from traditional birth attendants instead. From these themes we identified several salient barriers to both syphilis testing and treatment, leading us to identify four critical and modifiable targets: [1] structural barriers to care seeking that reflect facility and provider policies that are not necessarily in line with governmental policies; [2] untapped opportunities to leverage alternative care providers (such as traditional birth attendants) to bolster ANC attendance and syphilis treatment uptake [3] improve health literacy around syphilis through education campaigns within the community to increase demand of timely syphilis screening and treatment and [4] sub-optimal patient-provider interactions in government run healthcare facilities.

**Conclusion:**

The study provides practical suggestions for enhancing syphilis awareness, engagement in care, and women’s experiences in ANC within a resource-limited, high-burden context. Future efforts should concentrate on extending syphilis education beyond clinics, utilizing Village Health Teams (VHTs) as trusted sources, and identifying barriers to improving healthcare quality for women, informing quality improvement programming.

**Supplementary Information:**

The online version contains supplementary material available at 10.1186/s12889-026-26263-1.

## Introduction

Although preventable and treatable, there is a tremendous disease burden attributable to maternal syphilis globally, which can lead to congenital syphilis, infant morbidity and mortality, and other adverse birth outcomes (e.g., pre-term birth, low birth weight) [1, 2]. Syphilis incidence varies by geographic region; the World Health Organization (WHO) African region experiences the greatest syphilis-attributable disease burden, accounting for 56% of maternal syphilis and 62% of congenital syphilis cases [3, 4]. The high prevalence of syphilis in this setting is particularly concerning in the context of a generalized HIV epidemic, as syphilis infection increases the potential for sexual transmission of HIV [5]. 

Left untreated, more than half (60%) of maternal syphilis cases result in adverse birth outcomes, including death of the fetus or infant [6, 7]. Fortunately, cost-effective treatment (benzathine penicillin G) is available globally and can be administered through injections during a single clinic visit (for early syphilis) or over a course of three weekly visits (for late-stage syphilis infection) [8, 9]. Still, global syphilis screening rates among women who attend antenatal care (ANC) remain low (66%) [4]. Timely diagnosis and treatment are essential for successful treatment and prevention of congenital syphilis [1]. Given that roughly half of people with syphilis will be asymptomatic [1], universal screening of pregnant people is a critically important tool for identifying and treating syphilis infection and avoiding adverse pregnancy and birth outcomes. Given the synergism between syphilis and other sexually transmitted infections (STIs), such as HIV, screening is particularly imperative in settings with high prevalence of HIV among pregnant people (both for those already living with HIV as well as those at risk of infection) [10]. 

Extant research on the epidemiology of maternal and congenital syphilis in Uganda is limited with only a handful of studies providing recent prevalence estimates and considerable heterogeneity in these estimates. Syphilis prevalence estimates among pregnant women in Uganda range from 2.9 to 5.9% among pregnant women tested through ANC [11, 12]. Data from a hospital-based study in 2015 found a congenital syphilis prevalence of 3.8% among infants [13]. Uganda’s 2023 National Clinical Guidelines recommend syphilis screening as part of routine ANC and recommend dual HIV/syphilis screening in ANC settings for those who are not known to be living with either condition [8]. 

Although WHO and Ugandan national guidelines promote syphilis test-and-treat programming for pregnant people and their partners, and despite free provision of syphilis treatment to pregnant people attending government-run health facilities, challenges to timely testing and treatment in this population persist [14–17]. For example, while failure to link into ANC is associated with higher rates of congenital syphilis, the majority of poor pregnancy outcomes attributable to maternal syphilis actually occur among women who attend ANC but do not receive syphilis screening and/or treatment (i.e., missed screening opportunities) [14]. This low coverage may be due to several factors, including supply chain issues that reduce test and treatment availability [15, 17], reluctance to undergo testing and treatment, as well as low rates of partner treatment (leading to reinfection) [16]. A study in Western Uganda found a postpartum syphilis prevalence of 21.8% among women who had attended ANC but did not receive prenatal syphilis screening underscoring the syphilis burden being “missed” through current screening practices [18]. In addition, although nearly all Ugandan women (97%) attend at least one ANC visit during pregnancy, many delay seeking care until later in pregnancy, which increases the risk of syphilis attributable adverse birth outcomes that could have otherwise been prevented through identification and treatment prior to the end of the second trimester [19, 20]. 

To improve uptake of syphilis testing and treatment among pregnant women in Uganda, the barriers driving poor testing uptake in this population must be understood. There is a paucity of prior work exploring barriers to syphilis testing and treatment in Uganda, but two prior qualitative studies were identified [10, 21]. The first, among female sex workers, identified stigma (internalized, enacted by providers, and anticipated) as a salient barrier to requesting routine testing while wanting to remain healthy was identified as a facilitator to testing [10]. A second study in the general population of women identified several individual-level (fears around injection pain, concerns around partner notification, and limited knowledge about syphilis) and structural-level (access, time and work challenges, and clinic capacity) barriers to achieving adequate treatment coverage [21]. Beyond the two studies described, substantial gaps remain in understanding pregnant people’s attitudes and behaviors towards maternal syphilis screening and treatment, and how these attitudes and behaviors may serve as facilitators or barriers to testing and treatment uptake. To inform programming to overcome these barriers, the present study qualitatively explores perceptions of syphilis risk, transmission, testing, and treatment, as well as experiences accessing antenatal care, from the perspectives of currently pregnant people residing in communities across two Ugandan districts: Wakiso and Hoima.

## Methods

Reporting was guided by the COREQ checklist; the completed checklist is included as a supplemental file.

### Parent study and participant recruitment

Africa Medical and Behavioural Sciences Organization (AMBSO) is a not-for-profit research organization that was established in 2018 by a team of experienced Ugandan epidemiologists and biomedical researchers. The AMBSO Population Health Surveillance (APHS) Study has been described in detail previously [22]. In brief, APHS is an ongoing, longitudinal community-based open-cohort study in central (Wakiso District) and mid-western (Hoima District) Uganda that is currently in its fifth consecutive round of data collection. The cohort is conducted across six communities, selected for attributes that collectively enhance generalizability to the broader Ugandan context. Setting itself apart from other ongoing Health and Demographic Surveillance Sites (HDSSs) in Uganda, APHS is the first cohort to include urban sites in addition to rural and semi-urban sites.

Feasibility of Antenatal Syphilis and HIV Point of Care Testing to Prevent Perinatal Transmission in Uganda (FASTOM) is a nested study within APHS aimed at addressing gaps in knowledge of the scope of maternal syphilis and dual syphilis and HIV infection in Uganda. This mixed-methods study involved secondary analysis of APHS data and primary qualitative data collection with antenatal care providers (*n* = 20 interviews) and pregnant and postpartum women (k = 10 focus group discussions [FGD], *n* = 82) working and residing in APHS study communities. Sample size for the qualitative component was guided by the concept of saturation in salience, with data collection continuing until no new, substantively meaningful themes emerged across groups. Data from this study generated rich findings too expansive to cover in a single manuscript. Therefore, the present manuscript focuses specifically on data gathered during the FGDs with pregnant people to understand their perceptions of congenital syphilis, the importance of syphilis screening, and experiences receiving syphilis screening and treatment. Provider perspectives and quantitative findings will be explored in companion papers [23].

Participant recruitment for the present study was passive and relied on convenience sampling, with recruitment flyers hung in ANC clinics across six communities located in Hoima and Wakiso districts in Uganda. These clinics represent a subset selected from the six APHS study communities. While no prior relationship existed between the interviewers and participants before study recruitment, we recruited specifically in communities that participate in the APHS because AMBSO has well-established research and clinical ties in these locations, bolstering participant confidence and trust in the research team. Eligibility criteria were as follows: (1) adult women (aged 18 years or older) (2) having resided in either Wakiso or Hoima for at least 6 months; (3) currently pregnant or up to 12 months’ postpartum; and (4) having a phone or some other way of being contacted. Women who indicated interest in the study were screened and invited to attend one of the focus groups occurring in their community.

### Data collection

Experienced qualitative interviewers (male and female trained research assistants at AMBSO) conducted our FGDs. Participants were informed by the research assistants that they were public health researchers with AMBSO conducting a study on perceptions of antenatal syphilis and had no role in clinical care or service delivery. All data collectors were fluent in English and the native languages of Luganda and Runyoro, held a bachelor’s degree and had received training in qualitative methods and research ethics. Discussions were conducted in private, conveniently located spaces within the study communities that were familiar to participants, with only participants and trained research assistants present. Discussions were guided by a semi-structured interview guide (see Supplemental File 1) that explored topics related to syphilis knowledge and attitudes, care-seeking norms, and experiences attending antenatal care and receiving syphilis screening. Written informed consent was obtained from all study participants prior to data collection. Participants were compensated 10,000 Uganda shillings (UGX) (~$3 USD) for their time and 5,000 UGX ($1.50 USD) for transportation. FGD participants were also provided refreshments. We conducted a total of ten focus group discussions for an analytic sample of 82 participants. Focus groups ranged in size from four to ten participants and ran from 74 to 227 min (mean duration = 130 min). All focus groups were audio-recorded with participant consent.

This study was approved by the University of California, Los Angeles’ Human Research Protection Program (IRB#22–000928), Clarke International University (CIUREC/059) and Uganda National Council for Science and Technology (SS4468).

### Data analysis

The primary analytic objective of the present paper was to understand women’s experiences surrounding seeking ANC care to identify factors that might facilitate or prevent use of ANC services and/or STI screening and treatment. We restricted our focus to the FGDs with pregnant people to disentangle the perspective and experiences of women from that of providers.

The study employed an interpretive thematic analysis approach using both inductive and deductive coding. Focus group discussion recordings were translated and transcribed, then uploaded into Dedoose software for analysis. These transcripts were analyzed alongside debrief fieldnotes recorded by research assistants immediately following each focus group discussion. Codebook development was iterative and utilized both deductive and inductive approaches. Translated transcripts were reviewed in real time following a thematic and interpretive process, which informed the first draft of the codebook. A subset of transcripts was then double coded (10% of the total number of transcripts from the FGDs and IDIs, *n* = 3) by two reviewers (APM, NS) and code applications were reconciled, informing revisions and additions to the codebook to improve clarity. Following this process, one additional transcript was double-coded. When the two coders determined that coding consensus had been achieved, the remaining transcripts were subsequently coded independently. After all data were coded, the broader team came together to identify emergent themes. These themes then served as the basis of memoing, with three researchers (APM, NS, AW) reviewing relevant coded excerpts and summarizing thematic results. Finally, the memo results and interpretations were discussed by the larger team to reduce researcher bias. Rather than presenting a formal coding tree, findings are organized and reported by higher order thematic domains derived through this analytic process, consistent with an interpretive thematic approach.

Throughout the study design, steps were taken to promote trustworthiness as defined by Lincoln and Guba [24]. To promote transferability of our findings to our target population of interest (pregnant people), we utilized purposive sampling, open-ended questions, and a semi-structured interview guide. To improve the dependability of our results, we ensured that our codebook development was systematic and resulted in a final codebook with clear and discrete codes that could be consistently understood across coders. To promote confirmability, we utilized a reflexive and team-based approach to analysis. Finally, to improve credibility, our trained data collectors probed participants to elicit thick description, and we relied on peer debriefs (our study team is comprised of several Ugandan researchers) to check researcher bias.

## Results

Results have been organized into four thematic domains that informed study recommendations: (1) Barriers to optimal care engagement (2), Preferences for care outside of the healthcare setting (3), Community perspectives on syphilis risk and treatment, and (4) Poor experiences seeking ANC care at healthcare facilities.

### Barriers to optimal care engagement

#### ANC attendance

Participants described logistical, economic, and interpersonal barriers to appointment attendance. Wait times were generally long, with women spending most of the day at the clinic waiting for their appointment. Travel to and from clinics was also time-consuming and frequently associated with a cost if women had to hire a ride. Finally, the poor treatment many women reported receiving served as a major deterrent. Most women described being charged for aspects of their ANC at public facilities, despite these services being intended to be free, which created an economic barrier to care for many.

*Some mothers are living in absolute poverty; you will find that these mothers don’t have the listed requirements to go for antenatal care at a health facility*,* for instance “a leesu”… a bed sheet like cloth laid on a checkup bed in antenatal rooms before a health worker attends to you*,* so such mothers decide to shy away from going for antenatal care for fear of shame and embarrassment*,* and they end up giving birth at home. -Pregnant woman*,* Hoima*.

In instances where women needed to request financial support from their partners, be it to cover these transportation costs or to support the purchase of items at the clinic (such as a mama kit or prescription) partner refusal added another layer of difficulty.

*As a pregnant woman*,* there are times when you need something*,* say it is required at the health facility*,* when you request assistance from your partner*,* he will tell you that he doesn’t have money*,* some men are very violent*,* and he may end up fighting you. -Pregnant woman*,* Hoima*.

#### Syphilis treatment

A nearly universal barrier specific to syphilis treatment was fear of pain due to the penicillin injections. Some participants mentioned that, alternatively, an oral treatment was available, but this option was not free, and this created a financial barrier to treatment initiation or completion for some. Others indicated that their facility wouldn’t treat them for syphilis without the male partner being present, a barrier many women struggled to overcome since ANC visits were long and typically during the workday.

*Although the treatment is free*,* many people are terrified of the injection. Following that*,* there are rules governing the treatment*, e.g.,* syphilis treatment is not given to an individual but rather to a couple. Men have poor health-seeking habit. All in all*,* treatment is available at government aided facilities*,* and it is free of charge. Every time the husband refuses to take treatment*,* the woman endures the disease. -Pregnant woman*,* Wakiso*.

In several of the focus groups, some of the women recognized that treating an individual without treating their partner introduced a risk of reinfection and described how this contributed to misinformation regarding the curability of syphilis.

*That is the reason some people think syphilis is incurable. Men have a poor health-seeking habits. A woman may get treatment*,* but if the spouse does not get treated*,* there is always reinfection. -Pregnant woman*,* Wakiso*.

### Care seeking patterns and preferences

Due to poor experiences seeking ANC care at healthcare facilities in the past, as well as the barriers described above, many women indicated that they preferred to receive their ANC from traditional birth attendants (TBAs) instead of through health facilities. TBAs were perceived as providing comparable care, being kinder, and being less expensive, and they therefore were frequently used for ANC and delivery. As one pregnant woman in Wakiso described, *“TBAs make mothers’ lives easier. They deal with mothers as they are*,* with or without the necessary requirements.*

*Because the TBAs are usually elderly women who know the pain of childbirth*,* they care and usually reassure pregnant women which eases childbirth. In a government aided health facility*,* where you hope to get all the help you need during childbirth*,* things are different; the health workers are rude and often shout at expectant mothers. -Pregnant Woman*,* Wakiso*.

Having previously had a successful birth with a TBA was a reason many women sought TBA care in future pregnancies. TBAs also seemed to be more popular in rural areas. Poor treatment by providers, as well as long wait times and costs associated with visits were also described as reasons for delaying care seeking until later in the pregnancy, particularly among women who had already had a prior successful birth.

### Community perspectives on syphilis risk and treatment

Generally, participants demonstrated a basic understanding of syphilis transmission, with much of this knowledge stemming from personal experiences or those of others within their community. Most women understood that syphilis is sexually transmitted between partners and recognized that condom use could reduce risk of transmission. However, challenges related to sexual agency in marriage were identified as significant barriers to condom use.

*Syphilis is spread sexually. You can’t deny having sex with your husband. However*,* if you are not married*,* you can choose the time of your sex and even use condoms. -Pregnant Woman*,* Wakiso*.

*[Reinfection] is the reason some people think syphilis is incurable. Men have a poor health seeking habits. A woman may get treatment but if the spouse does not get treated*,* there is always reinfection. -Pregnant Woman*,* Wakiso*.

Many women demonstrated knowledge of the risk of congenital syphilis, recognizing that if they were infected with syphilis during pregnancy, there was a risk of transmitting it to their baby. Additionally, most were aware that syphilis was associated with adverse health outcomes for the baby, such as stillbirth and miscarriage.

*When syphilis is severe in infants*,* it might impair the child’s mental development. -Pregnant Woman*,* Wakiso*.

There were, however, also misconceptions, such as the belief that perinatal syphilis transmission rate was 100% or that syphilis could be acquired from dirty toilet seats.

*Well*,* the consequences of syphilis during pregnancy that I know are that if a pregnant woman does not experience a miscarriage during her pregnancy when she is syphilis positive*,* she will then give birth to a baby who is syphilis positive. -Pregnant Woman*,* Hoima*.

*One of the factors that could have increased my risk of acquiring syphilis infection during pregnancy is using dirty urinals and dirty toilets*,* also wearing underwear which have not been put under sunshine to dry up fully. -Pregnant Woman*,* Hoima*.

The participants recognized and described numerous symptoms that women may exhibit if they have a syphilis infection, including showing no symptoms at all.

*I am aware of syphilis. When a pregnant woman has syphilis*,* it might be difficult for her to detect it since sometimes there are no symptoms. As a result*,* she may give birth to a kid who has a bad rash and subsequently develops wounds before realizing she has syphilis. -Pregnant Woman*,* Wakiso*.

Medical misinformation around syphilis was also identified and appeared, at least in part, to be rooted in the perceived ubiquity of syphilis in the study communities. Participants referred to syphilis as “a black disease” and “a local disease”. One participant in a Hoima focus group theorized that it unpreventable because it was genetically transmitted.

*Most people have syphilis in their blood so it’s not easy to cure the infection because it is genetically transmitted. -Pregnant Woman*,* Hoima*.

Not all women were aware that syphilis treatment existed or that it was safe to treat syphilis while pregnant. Some women believed that experiencing symptoms again after treatment was proof that syphilis was not curable. Additionally, several women indicated that effective syphilis treatments, such as herbs remedies, were available from traditional birth attendants (TBAs).

*Yes*,* it can completely heal..an example my child had syphilis*,* and some woman gave me the local herbs that I used on my child and the disease disappeared and has never reappeared. […] However*,* the only problem is that it’s hard to find the right person selling such herbs because most of them are just after money*,* so they fake herbs. -Pregnant Woman*,* Wakiso*.

This misinformation may partly result from limited access to health information beyond anecdotal sharing of experiences among women in their community and the knowledge provided at healthcare facilities coupled with high utilization of traditional birth attendants (as opposed to clinical providers) for antenatal care and delivery. Most women indicated that they and the broader community lacked the means to access online health information.

*The reason as to why it’s not common is that you may find […] that out of the 15 women 3 or 4 women have smart phones and so it will be hard for mothers to seek information about their health via internet. Also*,* sometimes they lack enough time to go on internet. -Pregnant woman*,* Wakiso*.

#### Relative prioritization of syphilis screening and treatment

Nearly all women felt that screening for syphilis was important, largely due to recognition it was treatable and if left untreated could harm the baby.

*To me*,* I also think HIV and syphilis tests are the most important because when you are found syphilis positive*,* they give you treatment early so that you don’t transmit it to your unborn baby and the same applies to HIV. -Pregnant woman*,* Hoima*.

Still, many women felt that syphilis was relatively less important than HIV, which was considered deadly, which impacted the overall perceived priority of syphilis screening in antenatal care.

While many pregnant people felt that screening for syphilis was important and recognized that syphilis was associated with adverse birth and health outcomes for mother and child, there were several women who indicated that they were not concerned with syphilis because of its perceived ubiquity in their community, the fact it was treatable, and its relative severity compared to HIV. Similarly, HIV was unanimously described as the most important prenatal screening test.

*I am not afraid of syphilis as a disease. Syphilis is way lower in ranks compared to HIV. When you give birth to a baby with HIV*,* they are required to take medication the rest of their lives unlike syphilis which is easily treatable even with local herbs. Or injections. Syphilis cannot be compared to HIV; syphilis is easily treated. -Pregnant Woman*,* Wakiso*.

Participants were able to name several community-based, research and academic organizations in their community that provided outreach services to pregnant people, including syphilis tests. Village Health Teams (VHTs) were also considered very important for community mobilization and sensitization efforts around health issues and participants indicated that VHTs should be engaged to further increase syphilis awareness, testing and treatment.

*[These organizations are] effective because they have engaged the community through radio talk shows*,* and community outreach*,* they facilitate peers who engage with community members who bring feedback from the community to the health facility. -Pregnant woman*,* Hoima*.

### Experiences seeking ANC care at healthcare facilities

Women generally felt that it was important to attend ANC, but a perceived unwelcoming environment, sub-optimal care, and poor bedside manner among providers were recurring themes in descriptions of public healthcare facilities.

*Pregnant women avoid health professionals who are rough*,* use abusive language and are harsh and who do not care for them while they are pregnant.-Pregnant Woman*,* Hoima District*.

*I was admitted to the facility because I had severe foot edema*,* yet I received no assistance at all. I did not receive any medicine or management advice for the edema. I received no explanation. I was sorry I spent the time and money traveling to the institution. These are some of the issues we encounter at medical facilities. -Pregnant Woman*,* Wakiso*.

In addition, participants in several focus groups narrated how women who attended ANC with their partners were given priority and seen more quickly, to incentivize partners to attend. Some women described how this adversely impacted their care seeking experience if their partners were unable or unwilling to attend with them.

*When you go for pregnancy care with your husband*,* they real work on you so fast and by the way*,* pregnant women who come with their husbands are always given the front seats and they come first in the queue. When you go alone irrespective of whether you arrived there early morning*,* they can work on you even at 3pm when they have finished working on those women who came with their husbands. -Pregnant Woman*,* Hoima*.

*I pleaded with the health worker since I had come with my husband at my previous visit. She allowed to attend to me but for only that visit. I was asked to bring my husband on the next visits and that I will not be offered antenatal care if I do not come with my spouse. -Pregnant Woman*,* Wakiso*.

Still, there were several women who indicated their overall ANC experience had been positive, highlighting diverse care seeking experiences.

*I receive antenatal care at a government health facility*,* but the health workers are polite and do not care whether you have money or not. They attend to all pregnant mothers in order of arrival. They ask for money only when a particular medication that you require is not at the facility. There is also an option where they write you the medications and you buy at a nearby pharmacy. -Pregnant Woman*,* Wakiso*.

## Discussion

Given the continued disease burden attributable to syphilis in sub-Saharan Africa and the limited research on contextual barriers to antenatal syphilis testing and treatment uptake, we qualitatively engaged pregnant people in two Ugandan districts (Wakiso and Hoima) to identify barriers that could serve as points of programmatic intervention. Our focus groups identified several salient barriers to both testing and treatment, leading us to identify four critical and modifiable targets: (1) structural barriers to care seeking that reflect facility and provider policies that are not necessarily in line with governmental policies (2), untapped opportunities to leverage alternative care providers (such as traditional birth attendants) to bolster ANC attendance and syphilis treatment uptake (3), low coverage of syphilis education opportunities within the community, and (4) suboptimal patient-provider interactions in government-run healthcare facilities. Recommendations for addressing these barriers are detailed below.

Through government facilities, specific care services (including ANC and labor and delivery), testing services (including syphilis testing) and treatments (syphilis treatments) are intended to be free. However, participants across our focus groups described being asked to pay for “free services”, which served as a deterrent for ongoing care engagement. A recurring example was that of the “mama kits”, a package of items that women need to deliver in a government facility. Theoretically, these kits (provided by the Ugandan Ministry of Health), which include basic sanitary supplies, such as plastic sheets, gloves, and gauze, should be provided to all women, free of charge at public and private health facilities [25]. However, individual facilities (and at times, individual providers) appear to charge a fee for the kits, making them inaccessible to some women. Because many participants perceived use of mama kits as a requirement for delivery at government hospitals, a resource intended to be free has inadvertently become a deterrent to ANC attendance and facility-based delivery.

Similarly, many of the women described receiving worse care when attending ANC visits without their partners, including longer wait periods and being denied syphilis treatment. While Uganda clinical guidelines do recommend testing and treating male partners, this is not a requirement for treating pregnant people [8]. There are benefits to engaging male partners in prenatal care, with studies suggesting that male partner engagement can lead to improved ANC attendance, greater odds of giving birth in a healthcare facility, and positive birth and health outcomes for mother and child [26–30]. However, intimate partner violence (IPV) is prevalent in Uganda, including among pregnant people [31], and can lead to a myriad of adverse health outcomes for mother and child [32]. Male partners who perpetrate IPV are less likely to attend ANC with their partners [26, 33–35]. While the intention at these facilities may have been to motivate partner involvement and prevent syphilis reinfection among partners, pressuring women to include male partners in their care and penalizing women who are unable to do so is not only a barrier to care and treatment, but it may also directly threaten their health and safety. Further training of provider and clinic staff around existing clinical guidelines, as well as greater oversight regarding resource distribution to patients, is needed to realign clinic practice with national policy.

Women in our study largely indicated that the preferred ANC providers in their community are TBAs and this preference is rooted in the fact TBAs overcome several barriers to care seeking. TBAs were viewed as kinder, more accepting, more affordable and more convenient. TBAs play a central role in maternal healthcare in LMICs worldwide [36] and for this reason, the World Health Organization (WHO) recommends that formal healthcare providers collaborate with and engage TBAs to improve women’s overall access to maternal care [37]. In contexts where interventions have been successfully implemented to support integration of TBAs into maternal healthcare maternal mortality rates have declined and neonatal outcomes have improved [38–40]. Further, several African nations have formally integrated TBAs into their primary health care, including Ghana, Zimbabwe, and Ethiopia. Given the preference for TBAs over formal healthcare workers expressed by participants in our study, establishing a collaborative relationship where TBAs can support the identification of women in need of syphilis testing and treatment and motivate them to seek care may serve as a critical resource for increasing the reach of testing programs. Education of TBAs on evidence-based syphilis treatment may also help to dispel myths that syphilis can be treated through homeopathic herbal remedies.

The need for greater syphilis literacy and an understanding of the importance of timely attendance at ANC in the community at large was another emergent finding. While many women demonstrated basic syphilis literacy, the belief that syphilis was either incurable or could be treated with homeopathic remedies came up in several focus groups as a reason for decreased advocacy for syphilis screening and biomedical treatment during pregnancy. Additionally, consistent with findings from qualitative work with men and women elsewhere in Uganda, while participants generally knew syphilis was sexually transmitted, misperceptions persisted. For example, consistent with prior work, some believed there was a genetic component, while others attributed transmission to dirty toilets [41]. Women indicated that limited access to online educational resources (due to low levels of smartphone and computer ownership) meant that healthcare providers were frequently the only source of education available. Given that many women do not attend ANC until later in pregnancy and may only attend a single session [42], increased opportunity for syphilis education outside of healthcare settings is needed. Timeliness of attendance at ANC has previously been identified as a barrier to routine ANC syphilis in sub-Saharan Africa [15]. Village health teams (VHTs) were identified as trusted resources in the community and suggested by participants as ideal points of contact for community-based knowledge dissemination. Use of VHTs to engage and educate community members was a successful strategy during the COVID-19 pandemic [43] and leveraging of existing community health worker infrastructure, also makes it a cost effective, low resource burden approach.

A final recommendation from our study revolves around the need to address participant perceptions of suboptimal patient-provider interactions in government-run healthcare facilities. Unfortunately, mistreatment of women by providers and healthcare facilities during childbirth is pervasive, globally [44]. Participants in our study indicated that interactions with ANC providers in their communities are primarily negative due to the provider bedside manner, and that this could serve as a deterrent for care seeking. These perspectives are consistent with a scoping review, which identified poor communication, denial of service (in the case of our study, due to partners not being at the appointment), rough examinations, and harsh language as characteristics of care in sub-Saharan Africa more broadly [45]. Consistent with the WHO vision to achieve a world where “every pregnant woman and newborn receives quality care throughout pregnancy, childbirth and the postnatal period” [46], efforts to improve quality of care for women and newborns are critical precursors to improving ANC attendance and the proportion of babies delivered by skilled birth attendants. However, while ongoing training opportunities that go beyond biomedical understanding and help them to view patients in a more holistic way will likely improve bedside manner, the reality is that healthcare systems in this setting are overextended, and many providers experience burnout that impacts their ability to provide the quality of care they may wish to provide [45]. Therefore, continuing to find ways to increase provider capacity by task-shifting wherever possible (such as engagement of VHTs and TBAs to provide education) is also essential to improving quality of care.

Collectively, the results of this study offer actionable recommendations for improving syphilis literacy and care and treatment engagement as well as the overall quality of women’s experience in ANC in a low resource, high burden setting. Although the present study was qualitative, limiting the generalizability of our findings, our purposive sampling frame, use of experienced data collectors, inclusion of participants from a range of community types and districts and iterative, team based analytic approach promote the transferability of these findings to the broader Ugandan community. It is also important to reiterate that the data presented here focuses on the perspectives of pregnant women and does not reflect provider perspectives. However provider perspectives are explored in a seperate recently published manuscript [23]. Future work should focus on strategies to extend education around syphilis prevention and treatment outside of the clinics (e.g., through community-based testing) and leverage the trust and recognition of VHTs as purveyors of health knowledge in this context. This type of outreach may also improve timeliness into ANC, facilitating compliance with WHO guidelines which recommend that syphilis screening ideally occur in the first trimester [anusc^47^]. A focus on identifying barriers to improving the quality-of-care providers can give to women in this setting is also needed to improve patient care seeking experiences.

## Supplementary Information

Supplementary Material 1.

Supplementary Material 2.

## Data Availability

Authors are willing to provide data to other researchers upon request. Since raw qualitative transcript data includes full narratives that may lead to participant identification, data sharing must be curated to meet the needs of particular research aims. All data requests can be submitted to the corresponding author [apmiller@sdsu.edu].
