# Supplementary material for: “As long as you are married you cannot protect yourself against syphilis”: qualitative exploration of syphilis risk perception and antenatal care seeking among pregnant women in Uganda
Source: BMC Public Health. 2026 Jan 27;26:657. doi: 10.1186/s12889-026-26263-1 (PMC12918089; doi:10.1186/s12889-026-26263-1)
Supplement: Supplementary file 1 — Supplementary Material 1. [file 12889_2026_26263_MOESM1_ESM.docx]

**Semi-structured Focus Group Guide for Pregnant Women**

[Scripted preamble]

1. **Ice Breaker**
   1. First, so we can all get to know each other a little, let’s go around and have everyone tell us what your due date is, or if you have recently given birth, the age of this child. You may also share the ages of any other children you have.
   2. What are the things about motherhood that excite you?
2. **Health Information Seeking**

Now I would like for us to talk about the ways women in your community get information about their health, overall and during pregnancy.

- 1. When pregnant women have questions about their health – in general or related to their pregnancy, where can they go for information? *Probe – The doctor, a friend, a family member, the internet, books?*
     1. [If not mentioned] How do women feel about seeking information from a health care provider or the health department?
  2. How common is it for women in your community to seek health information online?
     1. What devices do you use to connect to the internet? *Probe: Cell phone or computer? Is it easy to get internet access?*
     2. Which websites are do they use? *Probe: Website names, Blogs / names of blogs?*
     3. How do you tell if health information you find online is trustworthy?
  3. How do pregnant women prefer to get health information? *Probe – Newspapers, websites, women’s groups, radio shows, discussion with people, video, television, other written information (Pamphlets/brochures).*
  4. Have you ever downloaded an app that has pregnancy-related health information?
     1. If yes, which app did you download? How do you like it?

1. **Use of Health Services**

Now we’re going to talk about some things related to general health and pregnancy. Throughout the focus group, whenever we talk about healthcare, please keep in mind that we are referring to care *since becoming pregnant*. Also remember that any answers you give us will be kept completely confidential.

- 1. What are the biggest problems pregnant women face when trying to access healthcare? *Probe –Lack of ability to pay? Office is too far away / transportation problems? Cannot get an appointment? Do not have a doctor?*
  2. I know women seek pregnancy care at different locations and from different providers so each of your experiences is unique. That said, I would like to know your general feelings about the process of getting care while pregnant.
     1. How many pregnancy care visits do women usually have during a full-term pregnancy?
     2. How easy is it for pregnant women to schedule appointments with their health care provider?
     3. How long do women usually have to wait each time they go to a clinic for pregnancy care?
     4. How far along are most women (in weeks) when they attend their first appointment?
  3. When women have a problem with or a question related to their health, do they feel comfortable talking to any of their health care providers (about the problem or questions)?
     1. If yes, who? If no, why?? (nurses, physicians, midwifes)
     2. With which health professional do pregnant women spend more time?
     3. Are there any health professionals pregnant women try to avoid?

**IF NEEDED/APPROPRIATE – CONSIDER TAKING A 10 MINUTE BREAK HERE**

1. **Prevention Strategies**

During pregnancy care it is common to inform you how to make the best choices for you and your growing baby and to provide services for that at health care facilities. These services include informational materials that are given to promote healthy life styles, and activities that promote health and prevent diseases. For example, tests are often conducted during pregnancy care to prevent complications to your health and your baby’s health.

- 1. Have you been given any tests during your pregnancy to prevent some disease/health problem or to promote a healthier pregnancy?
     1. If yes, which tests?
     2. Within the health care team, who was the person responsible for asking you about or performing the tests?
     3. Did the person explain clearly what those tests are for and what test results mean until you can understand?
     4. Which is the test you most remember, and why do you think it made a higher impact on you?
     5. Were any of the test results provided on the same day? If not how did doctors deliver this information?
  2. Were you given any written information (e.g., brochures, fact sheets) about these tests? How was this information presented? Was it clear?
  3. Of all the tests you receive during pregnancy, which ones do you think are the most important? Why do you think that?
     1. *If syphilis testing is not specifically mentioned…*
        1. How important is testing for HIV during pregnancy? Why?
        2. How important is testing for diabetes during pregnancy? Why?
        3. How important is testing for syphilis during pregnancy? Why?
        4. How important is testing for anemia during pregnancy? Why?

1. **Knowledge**

Now we’re going to switch to talk about a specific health topic that some of you may have heard of. Just as a reminder, we are not going to ask you specifically about your experiences with this health topic. You can contribute to conversation as much or as little as you want, and all the information you share here will be kept completely confidential. Please do not discuss anyone’s comments or responses with anyone outside the group after the discussion. We are interested in what you and women in your community think. There are no right or wrong answers, we are just interested in getting your honest thoughts and opinions.

- 1. What do you know or what have you heard about syphilis? What about syphilis during pregnancy?

**SYPHILIS DEFINED:** So we are all on the same page, syphilis is a sexually transmitted infection that can cause serious health problems if it is not treated. Syphilis is divided into stages (primary, secondary, latent, and tertiary). There are different signs and symptoms associated with each stage.

- 1. Please tell me what you know about the consequences of syphilis during pregnancy on the health of your baby and on your health? *Probe (if not mentioned): Can syphilis be passed from a woman to her unborn baby?*
  2. If a pregnant women is infected with syphilis, what do you think the benefits are to her of being diagnosed during pregnancy? What are the benefits for her baby? At what time during the pregnancy (first, second, third trimester) do you think she should be tested?
  3. From your recollection, have you been tested for syphilis during a routine pregnancy care visit during this pregnancy?
     1. How were you tested?
     2. How did you feel about it?
     3. Were your questions answered (if you had any)?
  4. Before women get a syphilis test during pregnancy, do you think they can tell if they have the infection?
     1. What are some symptoms of syphilis?
     2. Do you think it’s possible that a woman might experience no symptoms even if she has syphilis?
  5. Do you think a pregnant woman with syphilis can receive treatment to cure the infection (while she is pregnant)?
  6. How can people protect themselves from getting syphilis or spreading it to others?
     1. Do you think it’s possible for a woman to be treated for syphilis but then be re-infected because her partner also has syphilis?
  7. Do you feel that women in your area are at higher risk of getting or having syphilis, compared to women throughout the rest of Uganda? What about elsewhere in sub-Saharan Africa?
  8. If available, would a syphillis test that you could get results and start treatment the same day be appealing to women in your community?
  9. What do you think are the most important messages for women like yourself to hear about the benefits of being tested for syphilis and receiving treatment if she is positive?

**Conclusion:** I would like to thank you all for your participation in this focus group! I appreciate the time you have taken to answer all of my questions and share your feelings with me and the group. I have learned a lot from you. If you are interested in receiving more information, we brought informational brochures about healthy pregnancy, STI testing including syphilis, and contacts you can reach out to. Please feel free to pick up any materials you would like. Before we end, do you have anything else you would like to share or any questions you would like to ask me? Thank you!
